# Neuroimmunology and (Epi)Genetics in Depressive Disorders

**DOI:** 10.3390/jpm11070670

**Published:** 2021-07-16

**Authors:** Piotr Gałecki, Katarzyna Bliźniewska-Kowalska, Michael Maes, Kuan-Pin Su

**Affiliations:** 1 Department of Adult Psychiatry, Medical University of Lodz, 91-229 Lodz, Poland; piotr.galecki@umed.lodz.pl; 2 Department of Psychiatry, Faculty of Medicine, Chulalongkorn University, Bangkok 10330, Thailand; dr.michaelmaes@hotmail.com; 3 An-Nan Hospital, China Medical University, Tainan 709, Taiwan

Depression causes individual suffering, loss of productivity, increased health care costs and high suicide risk. Despite the fact that depressive disorders are a serious brain dysfunction, their etiopathogenesis is not yet fully understood. Only 60–70% of patients suffering from Major Depressive Disorders (MDD) respond to the standard antidepressant treatment, which means that Treatment Resistant Depression (TRD) can be as prevalent as 1/3 of patients with clinical depression [1]. Hence, it is essential to continue searching for possible pathological mechanisms and potential contributory factors to TRD in order to find new, effective and safe therapies.

There is strong evidence that major depression (MDD) is a neuro-immune and neuro-oxidative disorder with increased levels of pro-inflammatory cytokines [2,3].

Dysregulation of the immune system is recognized as one of the most important contributory factors to TRD [1]. No wonder, then, that a serious question arises: whether specific interventions such as dietary modification can influence “psychoneuroimmunity” to help fight mood disorders. Activated immune-inflammatory pathways, dysregulated hypothalam-ic-pituitary-adrenal axis (HPA axis), and psychological issues might serve as links between the pathophysiology of depression and obesity [4]. In addition, certain nutritional or nutraceutical approaches, such as omega-3 fatty acids, might improve the neuro-immune and neuro-oxidative imbalance and therefore be beneficial for depressive patients [4,5].

According to the traditional monoamine theory of depression, most currently available antidepressant drugs were designed to inhibit monoamine reuptakes and increase the utilization of serotonin, noradrenaline and dopamine in the central nervous system (CNS). Now we know that monoamine deficiency is the result of more primary abnormalities. Monoamines are formed from tryptophan. However, in a situation of increased activation of the immune system, inflammatory factors cause excessive activation of IDO (indoleamine-2,3-dioxygenase), an enzyme present in microglia, astrocytes and neurons, catabolizing the transformation of tryptophan into the neurotoxic kynurenine (KYN), thereby reducing the availability of tryptophan for the production of serotonin. Kynurenine, in turn, influences the intensification of neurodegenerative processes [6].

Research on twins, families and adoptive children suggests the influence of genetic factors on the development of depressive disorders. The expression of a given feature is influenced not only by the sequence of DNA nucleotides, but also by epigenetic factors [7]. The expression of a given gene can be modified by external factors such as, for example, infections, stress and diet, and then be inherited.

Due to epigenetics, new research highlights the importance of early stages of development [8], including early childhood and the prenatal period, in the etiopathogenesis of depression. According to the neurodevelopmental theory, environmental factors affecting a pregnant woman may indirectly induce epigenetic changes in the fetus, increasing its risk of developing depressive disorders.

Regarding the biological hypotheses (e.g., neurogenic theory), several molecular factors, including those involved in neuroplasticity, might serve as biomarkers of affective disorders [9,10]. Some of these factors are associated with prognosis and therapeutic response. The most useful are those with a concentration that can be easily measured in the peripheral blood.

Further research into the biological basis of depressive disorders is necessary for their targeted, personalized treatment.
